# Structure-controlled asperities of the 1920 Haiyuan *M*8.5 and 1927 Gulang *M*8 earthquakes, NE Tibet, China, revealed by high-resolution seismic tomography

**DOI:** 10.1038/s41598-021-84642-7

**Published:** 2021-03-03

**Authors:** Quan Sun, Shunping Pei, Zhongxiong Cui, Yongshun John Chen, Yanbing Liu, Xiaotian Xue, Jiawei Li, Lei Li, Hong Zuo

**Affiliations:** 1 Key Laboratory of Continental Collision and Plateau Uplift, Institute of Tibetan Plateau Research, Chinese Academy of Sciences (CAS), Beijing, 100101 China; 2 University of Chinese Academy of Sciences, Beijing, 100049 China; 3 CAS Center for Excellence in Tibetan Plateau Earth Sciences, Chinese Academy of Sciences (CAS), Beijing, 100101 China; 4 Department of Earth and Environmental Sciences, Lehigh University, Bethlehem, PA 18015 USA; 5 Department of Ocean Science and Engineering, Southern University of Science and Technology, Shenzhen, 518055 China

**Keywords:** Natural hazards, Solid Earth sciences

## Abstract

Detailed crustal structure of large earthquake source regions is of great significance for understanding the earthquake generation mechanism. Numerous large earthquakes have occurred in the NE Tibetan Plateau, including the 1920 Haiyuan *M*8.5 and 1927 Gulang *M*8 earthquakes. In this paper, we obtained a high-resolution three-dimensional crustal velocity model around the source regions of these two large earthquakes using an improved double-difference seismic tomography method. High-velocity anomalies encompassing the seismogenic faults are observed to extend to depths of 15 km, suggesting the asperity (high-velocity area) plays an important role in the preparation process of large earthquakes. Asperities are strong in mechanical strength and could accumulate tectonic stress more easily in long frictional locking periods, large earthquakes are therefore prone to generate in these areas. If the close relationship between the aperity and high-velocity bodies is valid for most of the large earthquakes, it can be used to predict potential large earthquakes and estimate the seismogenic capability of faults in light of structure studies.

## Introduction

Earthquakes occur when the stored energy in the Earth’s lithosphere is suddenly released. Large earthquakes usually cause great hazards on natural environment and/or humans. There has been an obvious surge of great earthquakes with magnitudes ≥ 8.0 during the past decade with great diversity at various aspects^1^. The diversity of earthquake source is generally associated with the geometrical complexities of the fault systems, which are attributed to the heterogeneity of the dynamic rupture process^2–5^. Moreover, earthquake processes are significantly affected by the heterogeneity of mechanical properties of the fault zone, often represented conceptually as asperities^6–8^. Faults that are fully or partially locked by strong asperities breed great seismic hazard because the accumulated stress on the asperities are prone to be released through large earthquakes, contrasting sharply with the faults which are characterized by creeping deformation^9–11^. Seismological studies suggest the occurrence of strong earthquakes is closely related to the abnormal distribution of crustal velocity^12–15^. Therefore, studying the fine velocity structure around the source regions could shed light on the relationship between the velocity features and large earthquakes and furtherly the seismogenic mechanism of large earthquakes.

**Figure 1:**
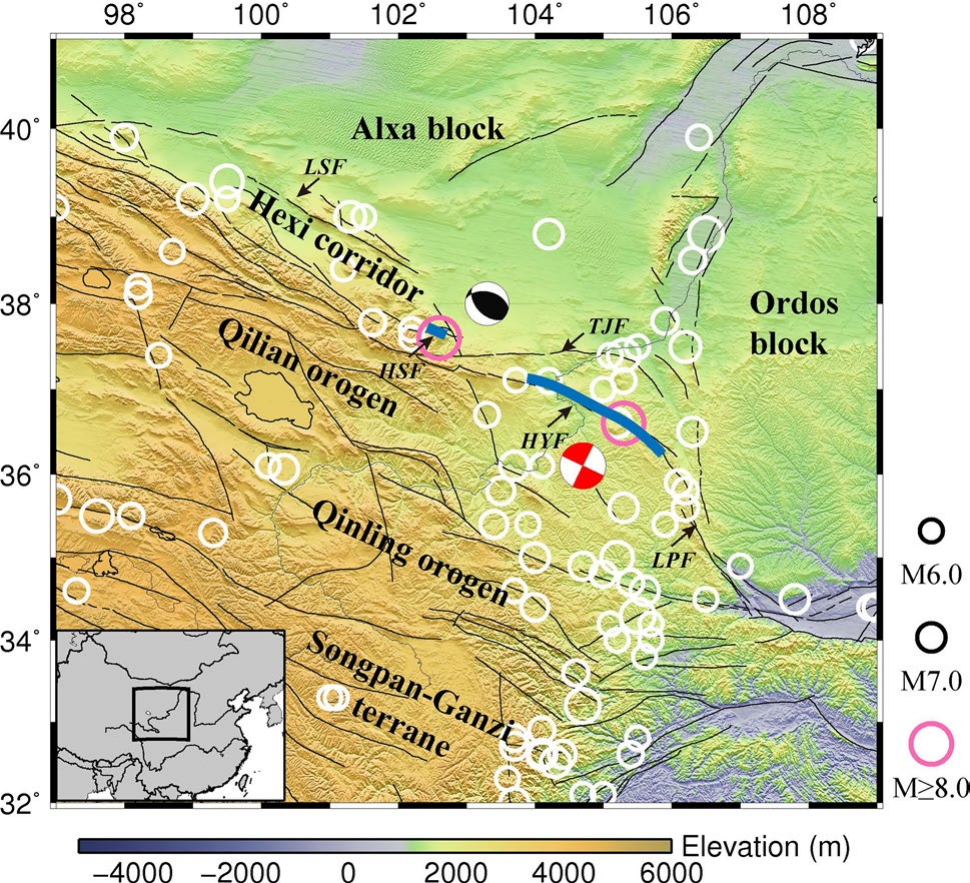
Tectonic background of the NE Tibetan Plateau and its surrounding areas. The white circles denote the locations of earthquakes larger than magnitude 6.0 and their sizes are proportional to magnitude. The two pink circles represent the 1920 Haiyuan *M*8.5 earthquake (on the right) and 1927 Gulang *M*8 earthquake (on the left), respectively. The two thick blue lines show the rough surface rupture zones of these two large earthquakes^23,24^, while their focal mechanisms are displayed by the two nearby beach balls respectively^23,25^. The black thin lines illustrate the main active faults^26^, among which the major ones are abbreviated as followings: LSF, Longshoushan fault; HSF, Huangcheng-Shuangta fault; TJF, Tianjingshan fault; HYF, Haiyuan fault; LPF, Liupanshan fault. The maps are created using Generic Mapping Tools (GMT)^27^ (v.4.2.1, https://www.generic-mapping-tools.org/).

Various studies have been conducted to explore the crustal structure of the large earthquake source regions and link the observations to mechanism of earthquake generation^16–18^. Besides, there have been a growing number of observations and numerical simulation studies on asperity^7,19–22^. However, so far there are few studies on the high-resolution local crustal structure surrounding the source regions of large earthquakes. To investigate the effects of structural heterogeneity on large earthquake generation, we explored the body-wave crustal velocity structure in the NE Tibetan Plateau where is high on seismicity (Fig. 1) and is therefore an ideal natural laboratory to study the seismogenic mechanism of large earthquakes. As one of the most active areas in continental China, numerous large earthquakes occurred there, including the 1920 Haiyuan *M*8.5 earthquake and 1927 Gulang *M*8 earthquake. These two earthquakes are extremely destructive and have led to a heavy loss on life and property. Though nearly 100 years have passed since the Haiyuan earthquake, there has not been enough research on these two large events due to the lack of data. Fortunately, in recent decades, abundant seismic data are increasingly available in the NE Tibetan Plateau, enabling us to obtain its high-resolution velocity structure and explore the seismogenic mechanism of the associated large earthquakes.

In this work, we improved the double-difference seismic tomography method and applied it to obtain a high-resolution three-dimensional (3-D) P wave and S wave velocities (Vp and Vs) model around the focal regions of the 1920 Haiyuan *M*8.5 and 1927 Gulang *M*8 earthquakes. Through analyzing their velocity features, we investigated the relationship between large earthquakes and velocity structure. Furthermore, we explored the seismogenic mechanism of large earthquakes in light of the asperity (high-V patches) which provides a guide for further seismic hazard assessment and associated disaster reduction. The detailed geometrical shape of fault plane is usually tough to depict, we therefore mainly discuss the seismogenic mechanism in terms of the structure features of the surrounding rocks.

## Data

**Figure 2:**
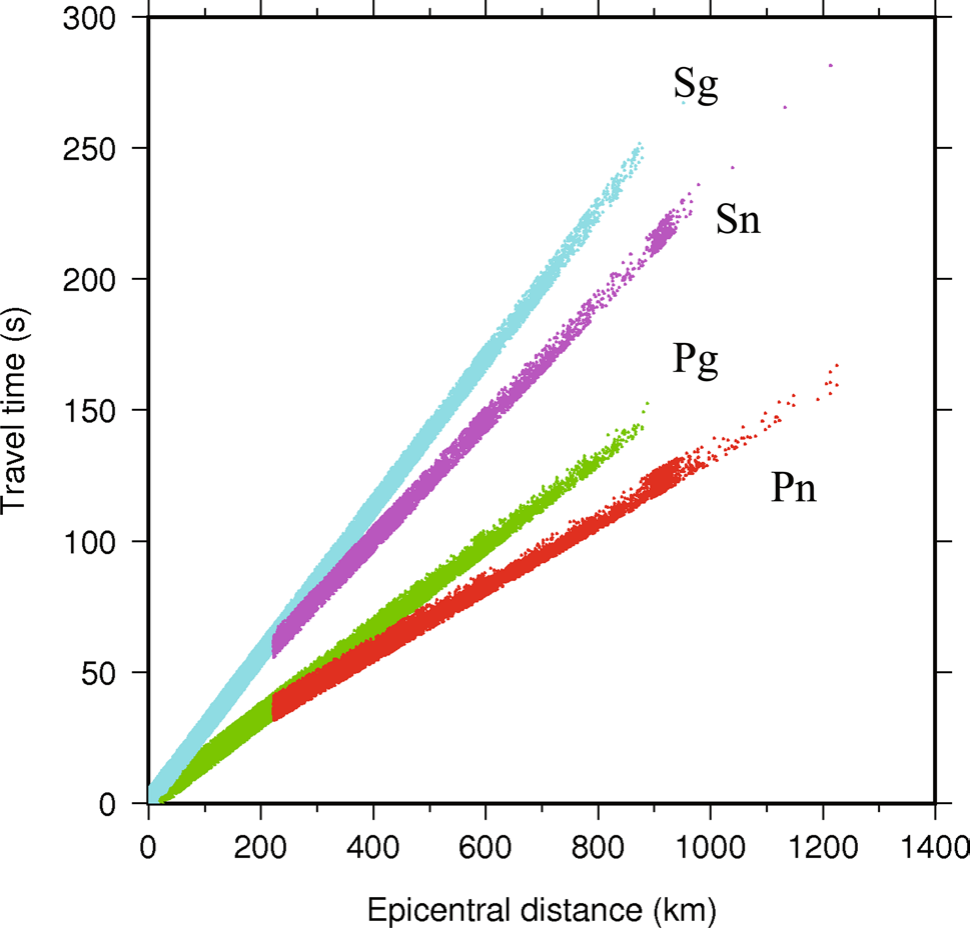
The distribution of travel times versus epicentral distance. The four different seismic phases are shown by different colors, with Sg (cyan), Sn (magenta), Pg (green) and Pn (red) from top to bottom.

Sufficient observed seismic data and additional picking of later phases are critical for high-resolution tomography. In this work, we collected large amount of travel time data in the study region from two sources: the history data from Gansu, Ningxia, Inner Mongolia and Qinghai provinces (1985–2008) and uniform data from National Earthquake Data Center (2009–2018) (http://data.earthquake.cn/)^28^. We carried out the following criteria on data selection: (1) all of the seismic hypocenters and seismic stations are distributed in the range of 32°–41°N latitude and 97°–109°E longitude. (2) Each event was recorded at least by 5 stations. (3) The epicentral distances for Pn and Sn waves are larger than 2°^29^. (4) The travel time residuals are less than 5.0 s. We finally obtained 325,829 Pg wave, 27,903 Pn wave, 311,646 Sg wave and 8,783 Sn wave from 34,997 local earthquakes recorded by 124 permanent and temporary seismic stations (Figure S1). The selected travel times of Pg, Pn, Sg and Sn wave exhibit universally linear relationship with the epicentral distance (Fig. 2).

## Results

### Resolution tests

Checkerboard test is a routine process for assessing the resolution and reliability of tomographic images. We assigned alternatively positive and negative velocity perturbations of 5% to the adjacent inversion grids of the initial velocity model. The synthetic travel times were calculated using the same ray distribution as the real data. Subsequently we applied our improved tomoDD method to obtain the recovered images and then compared with the original checkerboard model to check the recovery degree. Figure S3a and S3b shows the recovered checkerboard patterns for Vp and Vs tomographies around the source regions of Haiyuan and Gulang earthquakes with the lateral grid interval of 0.2°, respectively. On the whole, the checkerboard patterns were well recovered with resolution reaching to 0.2° above 15 km. Synthetic tests were also carried out to assess the reliability of the velocity anomalies, and these tests demonstrate that the main features of our tomographic results are well recovered at most of the areas (Figure S4 and S5).

### The velocity structure around the 1920 Haiyuan earthquake

**Figure 3:**
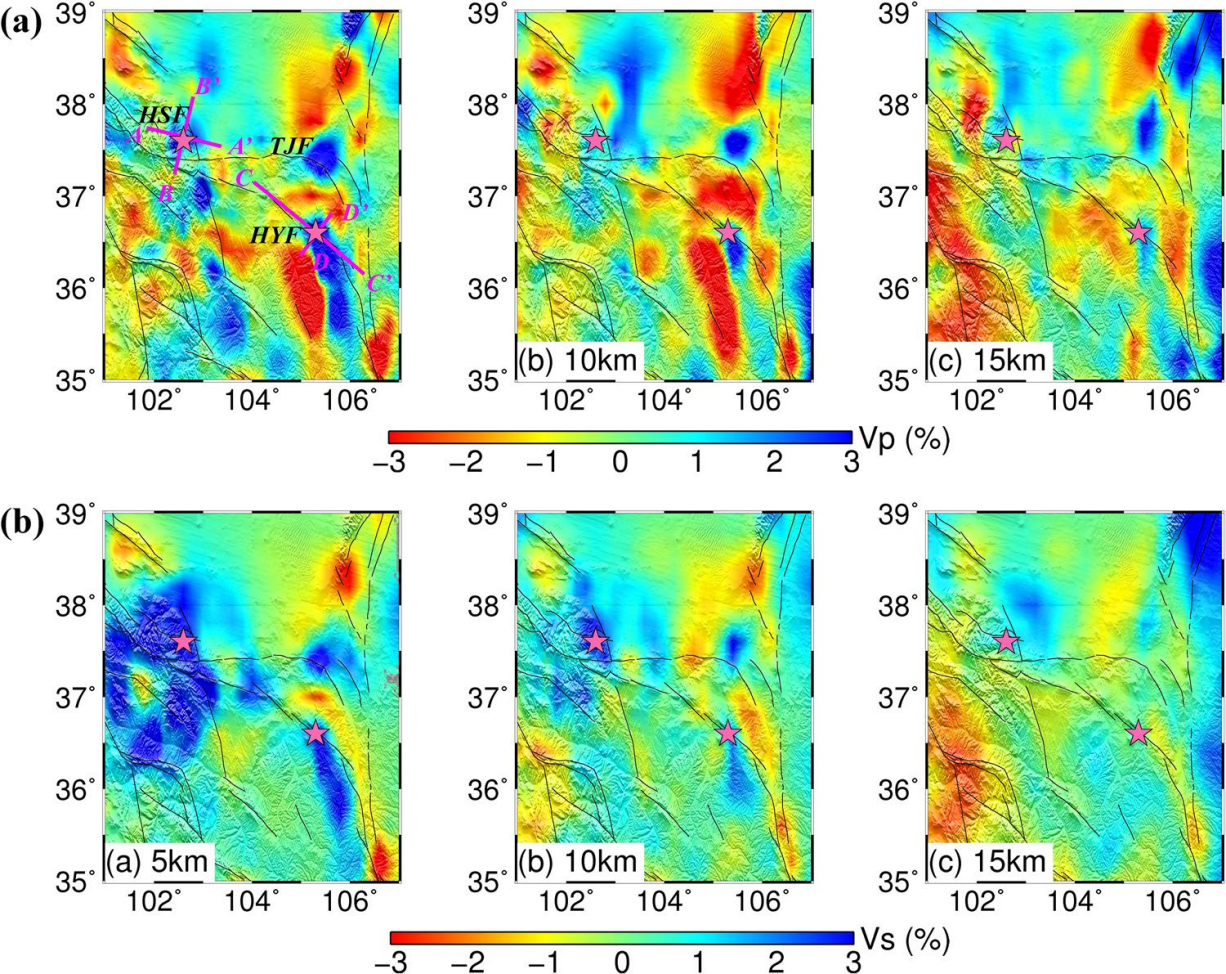
Tomographic Vp image (**a**) and Vs image (**b**) around the source regions of the 1920 Haiyuan and 1927 Gulang earthquakes, respectively. The magenta lines in Figure (**a**) represent the location of the four vertical tomographic profiles named AA′, BB′, CC′ and DD′ in Fig. 4.

**Figure 4:**
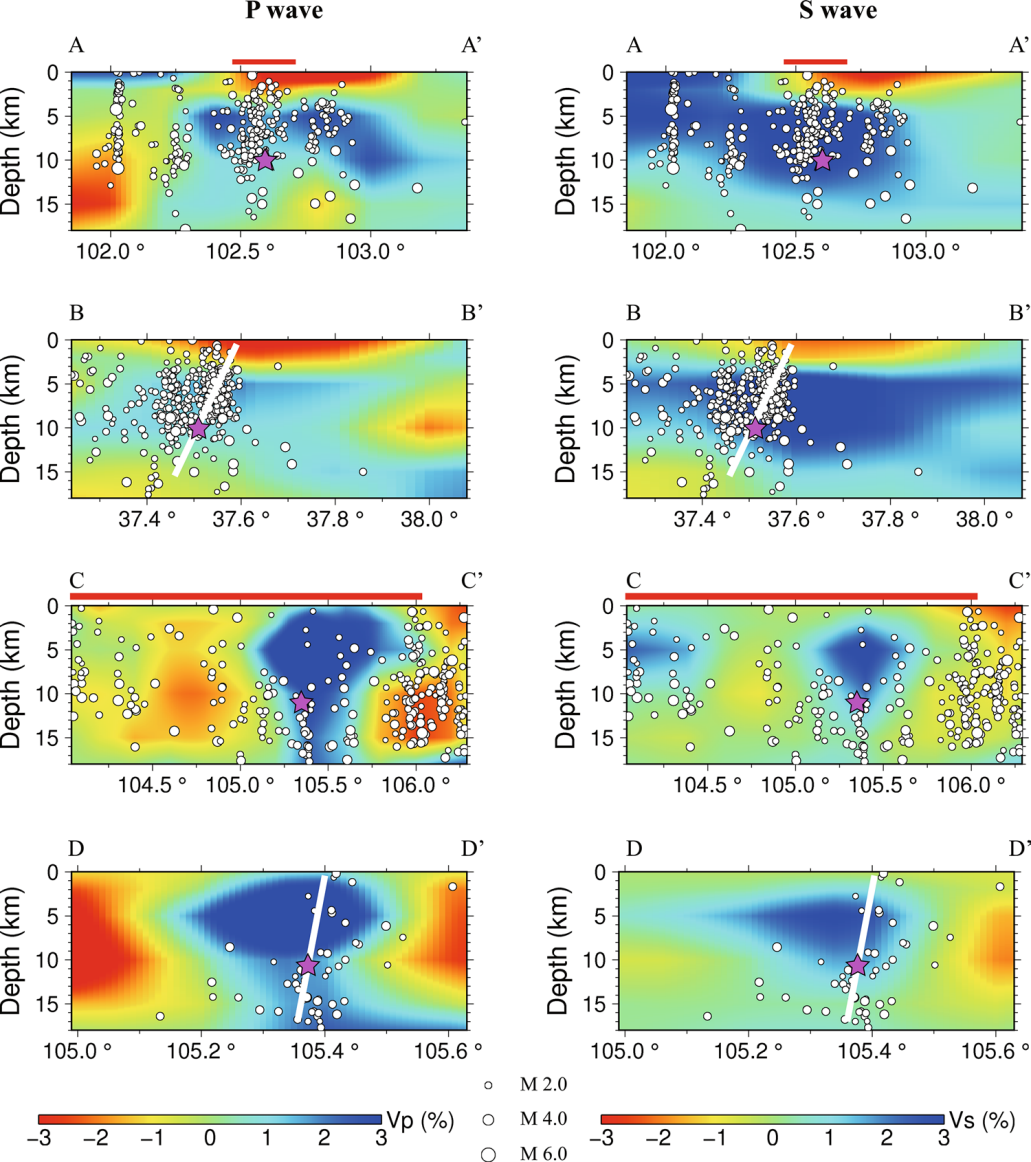
Vertical P and S wave tomographic images with relocated earthquakes. The thick red lines on the profile of AA′ and CC′ show the rough surface rupture zones of the two large earthquakes, and the white lines represent the major faults.

The 1920 Haiyuan *M*8.5 earthquake is one of the largest seismic events in continental China with an estimated hypocenter of longitude 105.3°E, latitude 36.6°N and depth 11 km^30^. It occurred on the Haiyuan fault, which is a sinistral strike-slip fault zone with a length span of ~ 1000 km and it connects the Qilian orogen to the west and the Qinling orogen to the east^31^. The fault rupture of this event is suggested to be about 220 km long^32^. Intraplate earthquakes in general occur within the brittle upper crust above 15 km. Figure 3a,b and 4c,d show the inverted velocity perturbations of P and S wave at different layers relative to the average velocity at each depth, respectively. The patterns of Vp and Vs images are generally similar to each other, although the size and amplitude of anomalies are slightly different somewhere which might be attributed to the relatively lower resolution of Vs maps. At the depth of 5 km, Haiyuan earthquake source region is dominated by southeastward elongated high-V anomalies surrounded by low-V anomalies, although the low-V anomalies in the Vs map are not as clear as that of Vp structure at the southwest of the source region. The high-V anomalies in Haiyuan earthquake source region extend downwards to the depth of 10 km, and are even visible at 15 km at Vp results but shrink in size and amplitude.

The tomographic velocity structure around the Haiyuan earthquake is comparable to the results from a more accurate deep seismic sounding study along longitude ~ 105.5°, which observed high-V anomalies above 25 km crossing Haiyuan fault^30^. The magnetotelluric study around the Haiyuan earthquake zone also demonstrated high resistivity in the focal area^33^, which is consistent with our results.

### The velocity structure around the 1927 Gulang earthquake

The *M*8 Gulang earthquake occurred on 23 May 1927 is another large event in the Haiyuan-Qilian fault belt after the 1920 Haiyuan earthquake. Its epicenter is approximately located at 37. 6°N and 102.6°E^34^. The surface-rupture zone is however much shorter, only 23 km long along the thrust^35^. From the high-resolution 3-D models obtained in this paper (Figs. 3a,b and 4a,b), Gulang earthquake source region has similar velocity patterns as Haiyuan earthquake and is dominated by high-V anomalies between 5 and 15 km. Especially on the Vs map, the high-V features are more obvious above 10 km. The focal area is surrounded by low-V anomalies, such as the Hexi corridor and southern area to the Gulang earthquake.

The significant high-V features which are noticeable above 15 km beneath the focal area of the Gulang earthquake have also been observed by a series of previous seismic tomography studies^36,37^, but our results show the anomlies in a much higher resolution due to the improved method and abundant data. The magnetotelluric sounding results show similar patterns with high-resistivity features above the focal area^38^. The gravity study also find high-density anomalies in the Gulang earthquake source region with high-V structures^39^.

## Discussion

The dominant finding in this paper is that the focal areas of the 1920 Haiyuan and 1927 Gulang earthquakes are both characterized by 3-D high-V anomalies above 15 km, which has also been revealed by former deep seismic sounding and magnetotelluric profiles^30,33,38^. Besides, the seismogenic faults exactly cut through the high-V bodies (Fig. 4). The high-velocity structures around these two large earthquakes are located around the Precambrian basement and/or Neoproterozoic to Early Paleozoic ophiolite sequences^40,41^.

Similar high-V anomalies around large earthquakes have been observed by many large earthquakes, such as the 1966 Parkfield M6 earthquake^14,16^, 1995 southern Hyogo M7.3 earthquake^18^, 2004 M6.8 Niigata–Chuetsu earthquake^42^, 2008 Mw7.9 Wenchuan earthquake^43^, 2010 Ms7.1 Yushu earthquake^44,45^, 2011 Tohoku-oki Mw 9.0 earthquake^46^, 2013 Ms7.0 Lushan earthquake^47,48^ and 2015 Mw7.8 Gorkha earthquake^15^. Therefore, it is probably a universal phenomenon that the high-V bodies in the source regions of large earthquakes represent the asperities in fault planes, which are essential elements for large earthquake generation.

**Figure 5:**
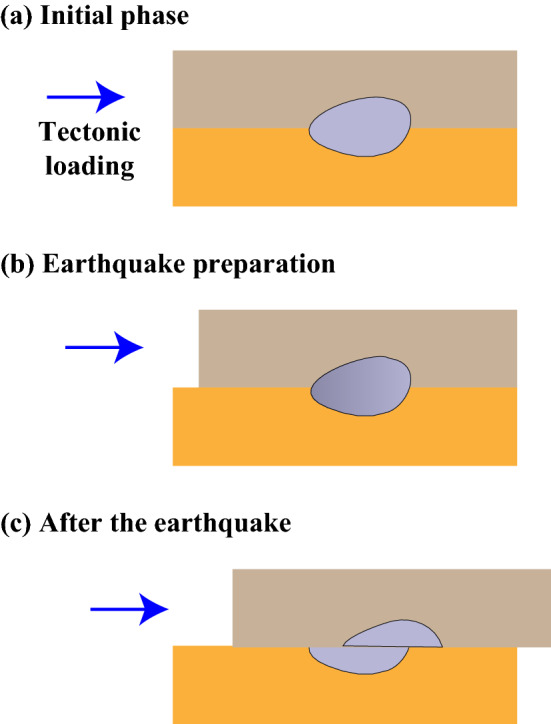
The cartoon illustrates the mechanism of preparation and generation of large earthquakes.

The asperity model^8,49^ has been widely adopted to explain the seismogenic mechanism of main shock of large earthquakes. The term asperity was originally defined as "unevenness of surface, roughness or ruggedness". Smooth surfaces, even those polished to a mirror, are not truly smooth on a microscopic scale. Surface asperities exist across multiple scales, and seismic asperity on a fault plane is at a macroscopic scale. Simply speaking, the asperity is a locked area before an earthquake (Fig. 5a), which has stronger localized mechanical coupling in the source region and is able to offer greater than average resistance to rupture^8^. The asperity will block fault from slipping or the fault slip will destroy the asperity in the fault plane. So the strength of asperity plays an important role in the initiation of fault slip. The stronger the asperity is, the more difficult the fault sliding is. Stress incessantly accumulates on the asperity in the interseismic period due to tectonic loading (Fig. 5b) until fault failure (Fig. 5c), and the maximum slip usually occurs at the asperity zone^8,50^. Generally, high-V bodies have high density, small porosity and then high strength^51^. Therefore, high-V bodies represent asperities and are the fundamental cause for the locking of fault planes, which finally contribute to the generation of large earthquakes.

If the close relationship between the aperity and high-V bodies is valid for most of the large earthquakes, it can be used to predict potential large earthquakes and estimate the seismogenic capability of faults in light of structure studies. In this work, there are clear high-V anomalies above 15 km crossing the Tianjingshan fault (Fig. 3), which suggests strong potential for large earthquakes. We then searched the history earthquakes from Chinese Earthquake Catalog and found the 1709 Zhongwei M7.5 earthquake (latitude 37. 4°N, longitude 105.3°E) just occurred there, which validates our conclusion. Therefore, if similar tomographic structure researches are carried out on all active large faults, it will be a great contribution to earthquake prevention and disaster reduction.

## Methods

The double-difference seismic tomography method (tomoDD)^52^ is developed from the double-difference hypocentral location method (hypoDD)^53^ and has the advantage to obtain the hypocentral locations and velocity structure simultaneously. TomoDD is suitable for local-scale tomography^43,54^. There are at least two issues need to be addressed when applied to large study areas for guaranteeing a reliable result. First, tomoDD simply sets the Moho discontinuity as a gradational rather a sharp interface, which inevitably biases the velocity structure around the Moho discontinuity. This problem is especially severe in our study area where the Moho depth contrast can be as large as 30 km between the thick NE Tibetan Plateau and the Ordos and Alxa blocks^55,56^. Second, tomoDD usually only uses first-arrival phases in the inversion, however there are plenty of later-arrival phases such as Pg and Sg waves in large epicentral distance over 200 km. These phases are essential for improving the coverage and crossing of ray paths in the middle-to-lower crust and thus could better constrain the velocity structure there.

To enhance the tomographic resolution, we improved the tomoDD method mainly in two aspects. First, detailed Moho depths beneath the study area are added as a prior information in the inversion, which help to obtain high-accuracy travel times of first-arrival Pn and Sn phases. The Moho results are mainly obtained from the dense stations of ChinArray^55^. For the areas out the coverage of ChinArray stations, the Moho results are collected from He et al.^57^. Second, we modified the ray tracing of tomoDD method for enabling to calculate the travel times of later-arrival Pg and Sg phases.

We set up a 3-D velocity model with horizontal spacing of 0.2° and ~ 5 km in depth according to the data density and checkerboard tests. Referring to the detailed 2-D velocity results from active-source seismic profiling in the study region^58^, we constructed the initial velocity model with the Moho depth taken into account (Figure S2).

The LSQR algorithm^59^ is used to solve the large and sparse system of observation equations which link the observed travel times to the unknown hypocentral and 3-D velocity parameters in tomoDD method. In order to minimize the instability during the inversion and balance model smoothness versus data fitting, smoothing and damping regularizations are adopted in the inversion. After testing different values of smoothing and damping parameters for finding the optimal trade-off point between the RMS of travel-time residual and the norm of the 3-D velocity model, the values of 10 and 600 were selected for the smoothing and damping parameters, respectively.

## Supplementary information

Supplementary information.
