# Supplementary material for: Structure-controlled asperities of the 1920 Haiyuan M8.5 and 1927 Gulang M8 earthquakes, NE Tibet, China, revealed by high-resolution seismic tomography
Source: Sci Rep. 2021 Mar 3;11:5090. doi: 10.1038/s41598-021-84642-7 (PMC7930187; doi:10.1038/s41598-021-84642-7)
Supplement: Supplementary file 1 — Supplementary information. [file 41598_2021_84642_MOESM1_ESM.docx]

*Scientific Reports*

Supporting Information for

**Structure-controlled asperities of the 1920 Haiyuan *M*8.5 and 1927 Gulang *M*8 earthquakes, NE Tibet, China, revealed by high-resolution seismic tomography**

**Quan Sun^1,2^, Shunping Pei^1,2,3^,** **Zhongxiong Cui^4^, Yongshun John Chen^5^, Yanbing Liu^1,2^, Xiaotian Xue^1,2^****, Jiawei Li****^1,2^, Lei Li^1,2^ & Hong Zuo^1,2^**

^1^Key Laboratory of Continental Collision and Plateau Uplift, Institute of Tibetan Plateau Research, Chinese Academy of Sciences (CAS), Beijing 100101, China. ^2^University of Chinese Academy of Sciences, Beijing 100049, China. ^3^CAS Center for Excellence in Tibetan Plateau Earth Sciences, Chinese Academy of Sciences (CAS), Beijing 100101, China. ^4^Department of Earth and Environmental Sciences, Lehigh University, Bethlehem, PA 18015, USA. ^5^Department of Ocean Science and Engineering, Southern University of Science and Technology, Shenzhen 518055, China. Correspondence and requests for materials should be addressed to S.P. (peisp@itpcas.ac.cn)

This Supporting Information contains the following results for complementing the statement in the main text: the distribution of seismic events and seismic stations (Figure S1), the schematic map showing how to construct the 1-D velocity model (Figure S2), the checkerboard testing results (Figure S3) and the maps obtained through synthetic tests (Figure S4 and S5).


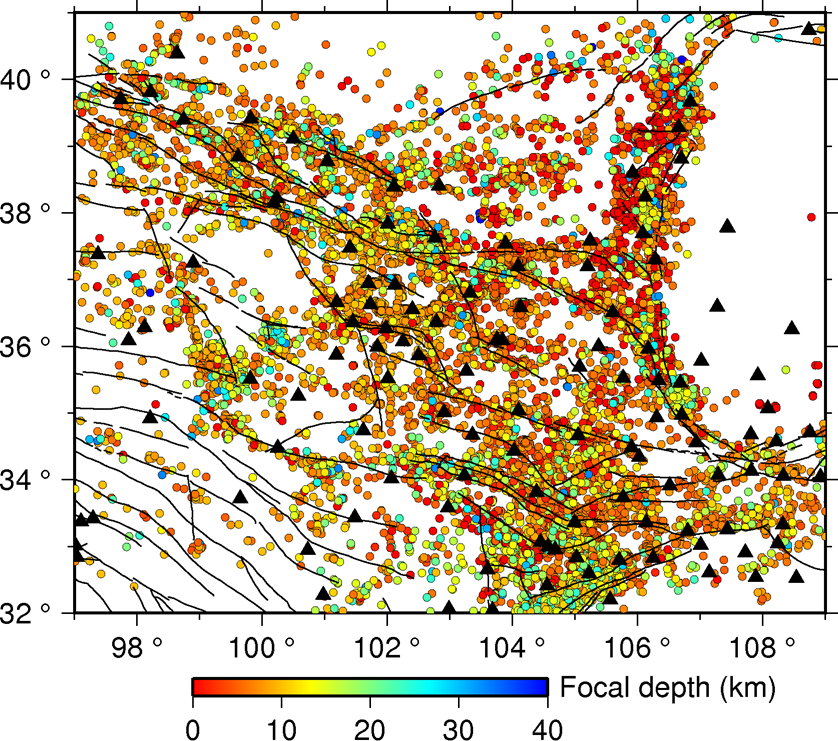


Figure S1. The distribution of seismic events and seismic stations used in this study. The colorful dots represent the earthquakes, and the black triangles show the seismic stations.


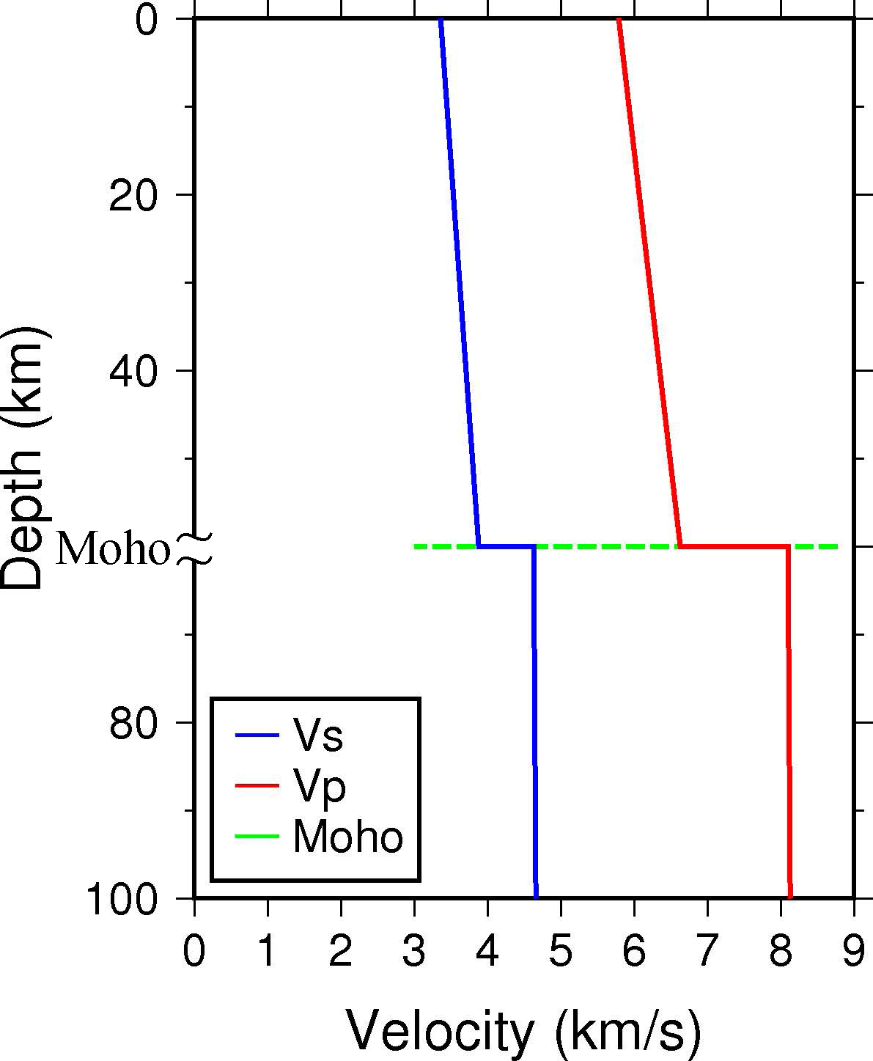


Figure S2. The schematic map shows how to construct the 1-D velocity model in the study. A Moho discontinuity with depth ranges from 36 km to 68 km^1,2^ in was used.


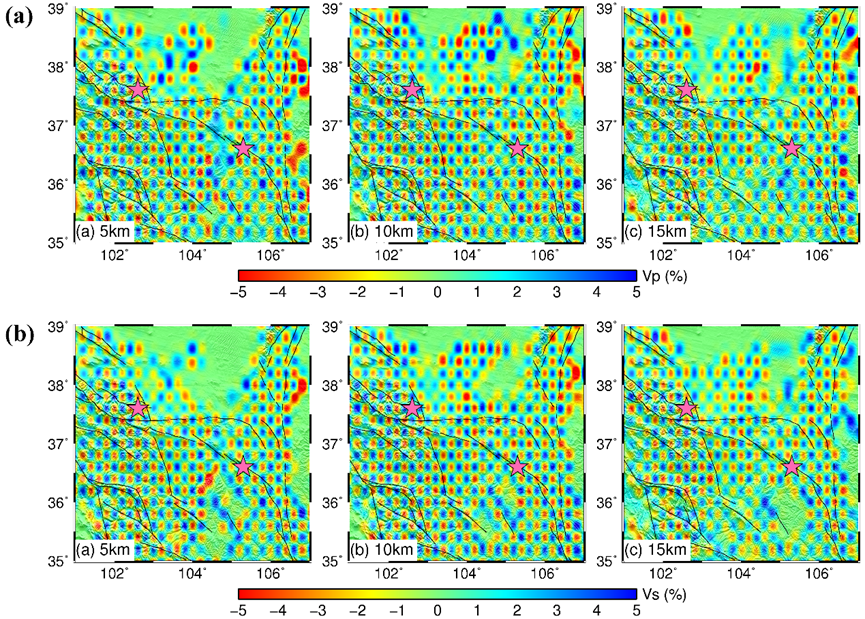


Figure S3. (a) and (b) show the P-wave and S-wave checkerboard tests around the source regions of the 1920 Haiyuan and 1927 Gulang earthquakes, respectively. The lateral checkerboard size is 0.2°.


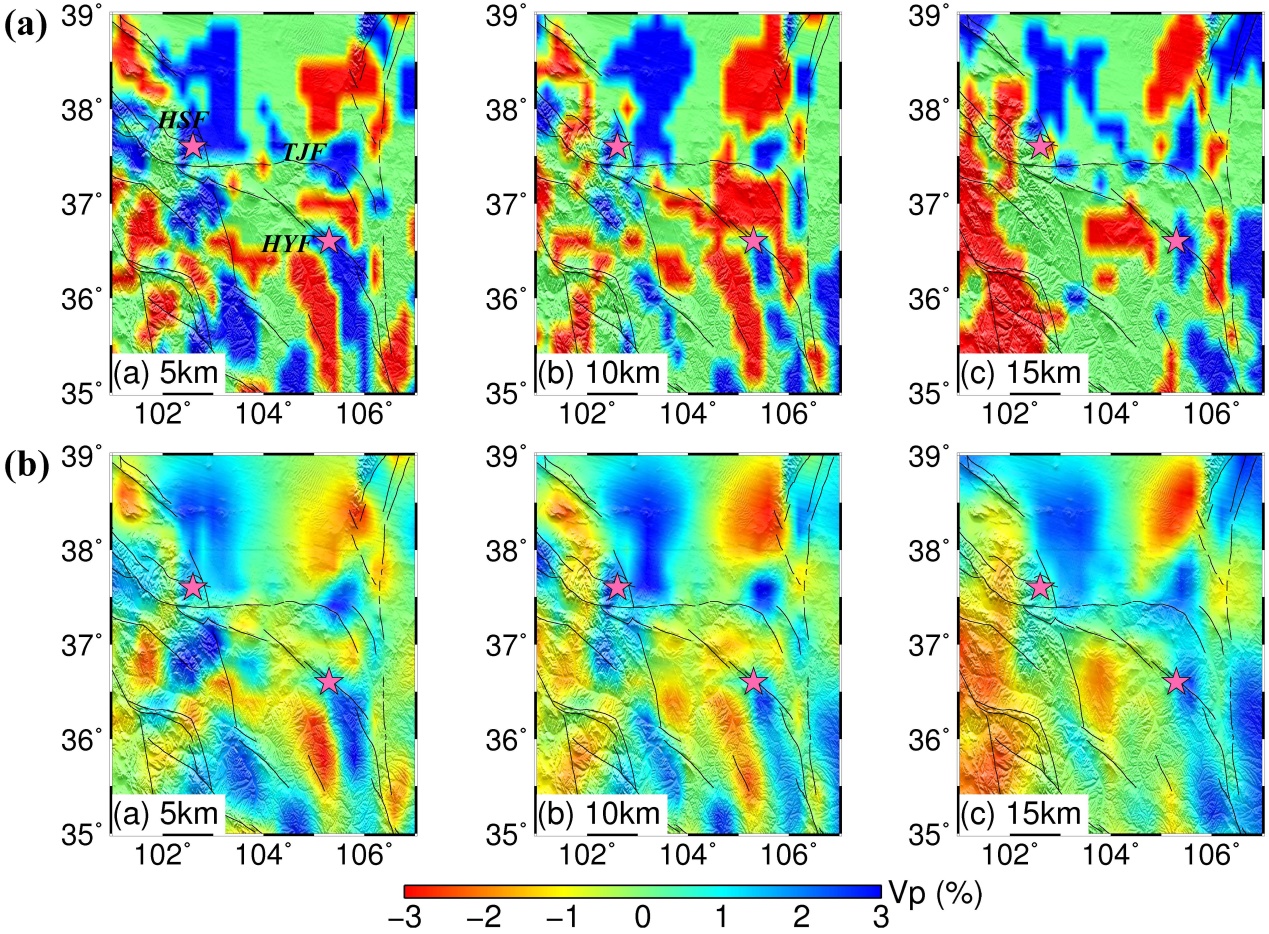


Figure S4 The results of synthetic tests for P wave tomography. The input model was built upon the obtained tomographic results of the study, but we changed the velocity anomalies as follows. For the grid nodes with velocity anomalies ≥ 1%, they are changed to 3%, while for those with velocity anomalies ≤ -1%, they are changed to -3%. For the grid nodes with velocity anomalies between −1% and 1%, they are changed to 0%.


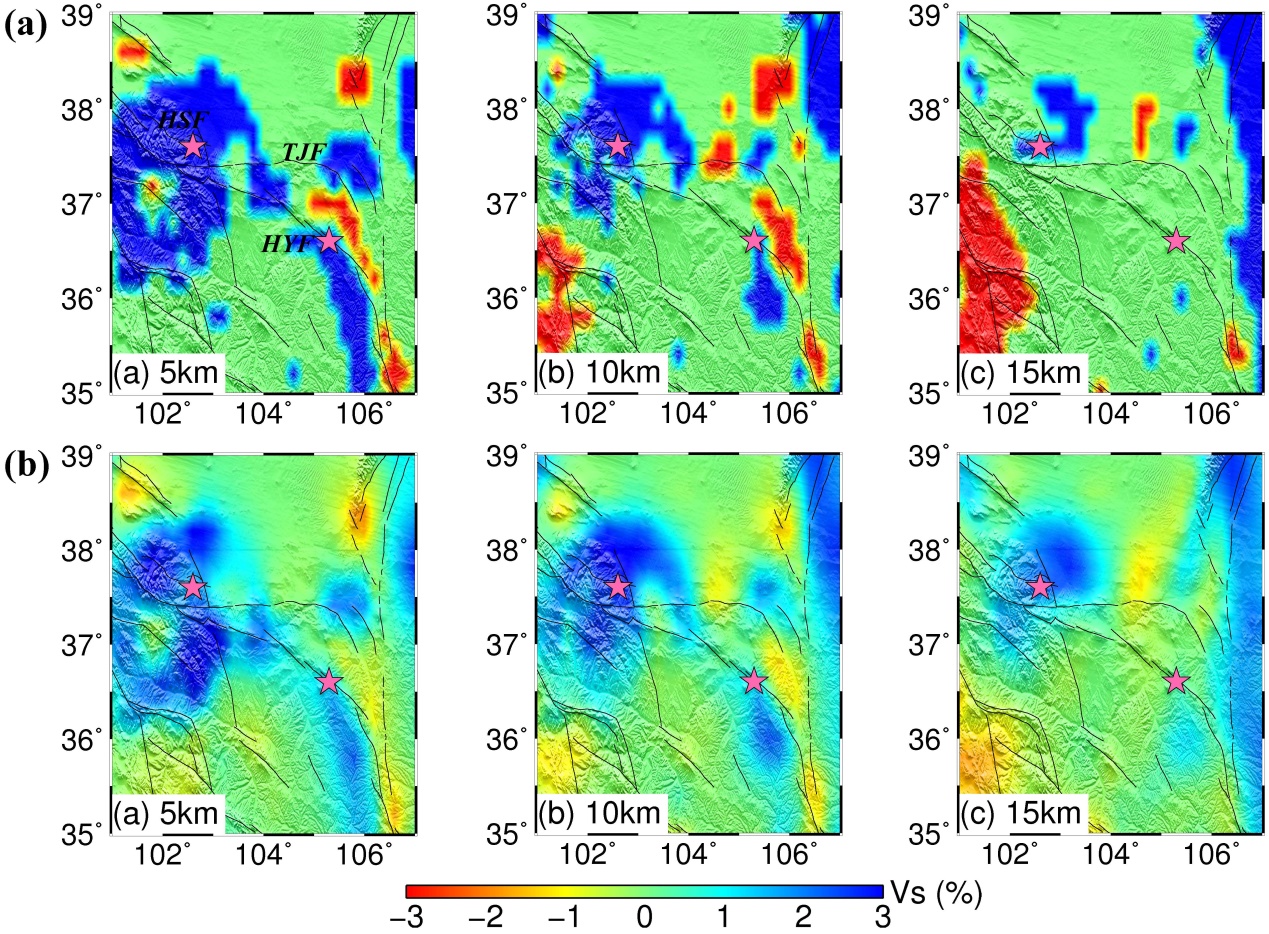


Figure S5 The figures are plotted same as Fig. S4 but for S wave tomography.
